# Supplementary material for: Resolving the full spectrum of human genome variation using Linked-Reads
Source: Genome Res. 2019 Apr;29(4):635–45. doi: 10.1101/gr.234443.118 (PMC6442396; doi:10.1101/gr.234443.118)
Supplement: Supplemental Material [file supp_gr.234443.118_Supplemental_longranger-2.2.zip › longranger-2.2.tar.bz2!/lr2.2-public/lib/stan/cmdstan/stan/doc/api/html/math_2matrix_2sd_8hpp_source.html]

Stan: src/stan/math/matrix/sd.hpp Source File


|  |  |
| --- | --- |
|  | Stan  2.5.0  probability, sampling & optimization |


- Main Page
- Related Pages
- Namespaces
- Classes
- Files

- File List
- File Members

All Classes Namespaces Files Functions Variables Typedefs Enumerations Enumerator Friends Macros Pages

- src
- stan
- math
- matrix

sd.hpp

Go to the documentation of this file.

1 #ifndef STAN\_\_MATH\_\_MATRIX\_\_SD\_HPP

2 #define STAN\_\_MATH\_\_MATRIX\_\_SD\_HPP

3

4 #include <vector>

5 #include <boost/math/tools/promotion.hpp>

6 #include <stan/math/matrix/Eigen.hpp>

7 #include <stan/math/matrix/variance.hpp>

8 #include <stan/math/error\_handling/matrix/check\_nonzero\_size.hpp>

9

10 namespace stan {

11  namespace math {

12

19  template <typename T>

20  inline

21  typename boost::math::tools::promote\_args<T>::type

22  sd(const std::vector<T>& v) {

23  stan::math::check\_nonzero\_size("sd(%1%)",v,"v",(double\*)0);

24  if (v.size() == 1) return 0.0;

25  return sqrt(variance(v));

26  }

27

34  template <typename T, int R, int C>

35  inline

36  typename boost::math::tools::promote\_args<T>::type

37  sd(const Eigen::Matrix<T,R,C>& m) {

38  // FIXME: redundant with test in variance; second line saves sqrt

39  stan::math::check\_nonzero\_size("sd(%1%)",m,"m",(double\*)0);

40  if (m.size() == 1) return 0.0;

41  return sqrt(variance(m));

42  }

43

44  }

45 }

46 #endif

variance.hpp

stan::math::sd

boost::math::tools::promote\_args< T >::type sd(const std::vector< T > &v)

Returns the unbiased sample standard deviation of the coefficients in the specified column vector...

**Definition:** sd.hpp:22

stan::math::variance

boost::math::tools::promote\_args< T >::type variance(const std::vector< T > &v)

Returns the sample variance (divide by length - 1) of the coefficients in the specified standard vect...

**Definition:** variance.hpp:24

stan::agrad::sqrt

fvar< T > sqrt(const fvar< T > &x)

**Definition:** sqrt.hpp:15

Eigen.hpp

check\_nonzero\_size.hpp

stan::math::check\_nonzero\_size

bool check\_nonzero\_size(const char \*function, const T\_y &y, const char \*name, T\_result \*result)

Return true if the specified matrix/vector is of non-zero size.

**Definition:** check\_nonzero\_size.hpp:33


---

[ Stan Home Page ]

*© 2011–2014,
Stan Development Team.*
